# Recent Breakthroughs in Supercapacitors Boosted by Nitrogen‐Rich Porous Carbon Materials

**DOI:** 10.1002/advs.201600408

**Published:** 2017-02-15

**Authors:** Mei Yang, Zhen Zhou

**Affiliations:** ^1^ School of Materials Science and Engineering Herbert Gleiter Institute of Nanoscience Nanjing University of Science and Technology Xiaolingwei 200 Nanjing 210094 China; ^2^ Tianjin Key Laboratory of Metal and Molecule Based Material Chemistry Institute of New Energy Material Chemistry Collaborative Innovation Center of Chemical Science and Engineering (Tianjin) School of Materials Science and Engineering National Institute for Advanced Materials Nankai University Tianjin 300350 China

**Keywords:** supercapacitors, N‐rich porous carbon, energy density, power desity, Li‐ion capacitors

## Abstract

Featured with unique mechanical, electronic and chemical properties, nitrogen‐doped carbon materials have become the research hotspot of energy storage. As electrode materials in supercapacitors (SCs), N‐doped carbons have demonstrated intriguing flexibility and superb performances in a wide electrochemical window, equipped with versatile properties as both cathodes and anodes for constructing high voltage devices. Compared with limited doping level, N‐rich and porous carbon materials (NPCs) are of great desire to release the restricted properties of N species and obtain high specific capacitances (>600 F g^−1^), pushing the energy density towards the battery level without scarifying the capacitor‐level power ability. In this Research News we firstly discuss the key factors influencing the performance of NPC electrodes to disclose related charge storage mechanisms. In addition, the trade‐off among N‐content, porous structure and electrical conductivity is involved as well as electrochemical behaviors in different electrolytes. Also, various progressive developments are highlighted systematically ranging from asymmetric to symmetric and hybrid configurations, covering both aqueous and non‐aqueous systems. Finally, some stubborn and unsolved problems are summarized, with prospective research guidelines on NPC‐based SCs.

## 1 Introduction

Carbonaceous materials are proposed as the best option for energy‐related applications because of natural abundance, facile accessibility and attractive properties, such as large surface area, high electrical conductivity, and excellent chemical stability.^1^, ^2^, ^3^ In this respect, they play critical roles as electrode materials in supercapacitors (SCs) and can be reversibly operated within a fairly large voltage window as well as versatility as both cathodes and anodes; however, their energy storage ability is largely limited because of the physical charge storage mechanism of electric double layer capacitors (EDLCs, <10 W h kg^−1^).^4^, ^5^ Therefore, it is desperately urgent to develop advanced functional carbon materials with enhanced electrochemical characteristics. Heteroatom‐ especially nitrogen‐containing carbon materials have attracted intensive attention as research hotspots in materials science over these years owing to their unique electronic, mechanical and catalytic properties, generating a crowd of exciting performances in related realms.^6^, ^7^, ^8^, ^9^

The doping of nitrogen has generated much improvement in physical properties of carbon materials, including conductivity and wettability. Firstly, substitutional introduction of more electron‐rich N into the C network could bring more electrons to the delocalized π‐system of carbon materials, which leads to increased electrical conductivity.^10^ For example, the configuration with N substituting for carbon atoms without destroying the sp^2^ network is beneficial to improve the electrical conductivity.^11^ Secondly, compared with common non‐polar carbons, N‐containing carbons are incorporated with more heterogeneous species and usually show better wettability.^12^, ^13^ These polar functional groups in the surface exhibit benign affinity to solvents with the same polarity, especially in the ubiquitously‐used aqueous systems. These hydrophilic species favor the wetting of electrodes and then the ion transportation; therefore, the capacitive performance would be enhanced. Moreover, the injection of extra electrons leads to the increase of density of states at the Fermi level, thus enhancing the quantum capacitance of graphene near the Fermi level when compared with the pristine one, which is proved by density function theory (DFT) computations and molecular dynamics (MD) simulations.^14^, ^15^ Besides, the nitrogen inclusion endows pristine carbon materials with additional redox reactions for pseudo‐capacitors, whose specific capacitances are several orders of magnitude greater than that of traditional EDLCs without sacrificing fast charging/discharging kinetics. For instance, three‐dimensional (3D) hierarchical porous N‐rich graphitic carbon materials achieved high specific capacitance of over 710 F g^−1^ and the novel symmetric SCs constructed with these materials in an aqueous acidic electrolyte could attain an energy density as high as 75 W h kg^−1^, which is over several times of EDLCs.^16^

Based on the intriguing mechanisms, N‐rich porous carbons (NPCs) have brought new breakthroughs to SCs and progressively pushed the energy density towards the battery level while keeping capacitor‐level power output, realizing high energy‐power integration to bridge the gap among current systems.^17^, ^18^, ^19^ First of all, compared with N‐doped carbon materials with limited doping level and specific capacitance (usually 200–400 F g^−1^), the development tendency towards N‐rich and porous carbon materials is of great desire to release the restricted properties of N species and obtain a much higher specific capacitance (>600 F g^−1^).^16^, ^20^, ^21^, ^22^ Construction of novel aqueous symmetric SCs with NPC materials exhibits competitive edges over the asymmetric counterparts by avoiding the universal energy/power imbalance at two ends. Consequently, the constructed symmetric SCs exhibit attractive energy density above 60 W h kg^−1^,^8^, ^16^, ^23^ outclassing the well‐studied asymmetric counterparts (20–40 W h kg^−1^) under the same power. Furthermore, fabrication of SCs in non‐aqueous electrolytes could proffer a high operating voltage of above 3.0 V, which considerably improves the energy density (>100 W h kg^−1^).^24^, ^25^, ^26^ Ultimately, lithium‐ion hybrid SCs based on NPC electrodes acquire the energy density level of lithium‐ion batteries (>200 W h kg^−1^) while maintaining the high power deliver of SCs, successfully realizing the high energy‐power integration.^27^

Therefore in this Research News, we particularly aim at recent progress in NPC‐based SCs, protruding the significance of NPCs for battery‐level supercapacitive performance. Fundamentals in NPCs and various progressive developments of NPC‐based SC configurations are highlighted systematically.^25^, ^27^, ^28^, ^29^ Revisiting the latest results would better understand N‐doping effects and shed light for the future development of high‐performance supercapacitors.

## 2 Key Factors Influencing the Performance of NPC Electrodes

### 2.1 Various N‐Species

**Figure 1:**
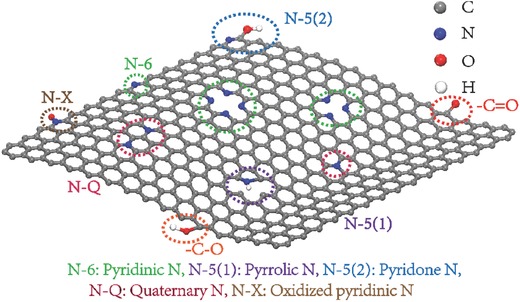
Various N‐containing functional groups in graphene.

As illustrated in **Figure** **1**, incorporation of nitrogen atoms into carbon honeycomb lattice commonly forms four types of functional groups: pyridine‐N (N‐6), pyrrolic/pyridone‐N (N‐5), quaternary‐N (N‐Q), and oxidized pyridine‐N (N‐X). N configurations with different electronic structures and hybridizations will introduce miscellaneous modifications on the properties of pristine carbon materials. Bonded with two sp^2^ carbon atoms, N‐6 and pyrrolic N atoms will contribute electron lone pairs into carbon conjugated systems, consequently inducing electron donor effects. Pyridone nitrogen atoms are also electron donating, whose pyridine nitrogen atoms are neighbored by a ring carbon‐hydroxyl group. On the other side, quaternary N atoms take the position inside an aromatic ring (graphitic N) with sp^2^ hybridization and fully saturated bonding, and further enhance the electrical conductivity of the carbon material. Arising from the thermally stable residues or adsorbent O_2_, oxygen‐containing groups always associatively exist along with NPCs, wherein pseudocapacitive contribution of some species is well established (e.g., related redox reactions of quinone oxygen groups are shown in Equation 2).^30^ The presence of O functionalities could enable participation in faradaic reactions and the combined effect between N and O groups is also beneficial for capacitance enhancement.

The miscellaneous properties of N dopants endow NPCs with favorable electrochemical behaviors different from common carbon materials, wherein triggered redox reactions always occur at doping sites or the adjacent carbon with altered electronic states. Generally, redox reactions are largely determined by the bonding structure within each N‐species. Note that pyridone nitrogen and N‐6 share similar ring structure; hence the electrochemical conversion between pyridone nitrogen and N‐6 is reasonable. On the contrary, since the structure change from five to six‐member rings only occurs at high temperatures,^31^ the transformation of pyrrolic nitrogen into N‐6 is extremely difficult under mild electrochemical conditions. Similarly, the rupture of N‐Q at the three‐fold “center” only occurs at high‐temperature gasification, indicating the inactiveness of N‐Q in electrochemical processes. As for N‐X, they typically represent the oxidized pyridine nitrogen atoms, which are supposed to be reducible through electrode processes.

**Figure 2:**
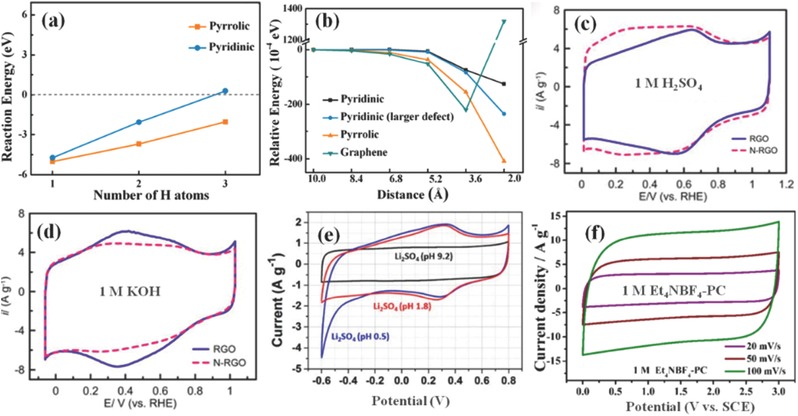
a) Reaction energy of H^+^ electrochemically adsorbed onto the surface of NPCs, and (b) the energy‐distance curves of different defect types. a,b) Reproduced with permission.^16^ Copyright 2015, Royal Society of Chemistry. Cyclic voltammetry (CV) of RGO and N‐RGO electrodes measured at 25 mV s^−1^ in 1 m H_2_SO_4_ (c) and 1 m KOH (d). c,d) Reproduced with permission.^30^ Copyright 2013, Elsevier. e) CV curves of OMFLC‐N in Li_2_SO_4_ electrolytes with different pH values at sweep rates of 2 mV s^−1^. Reproduced with permission.^8^ Copyright 2015, AAAS. (f) CV curves of NPC in 1 m Et_4_NBF_4_‐PC electrolyte. Reproduced with permission.^23^

In addition to the qualitative structural analysis, it is also indispensable to dig into the electron distribution of local C–N bonds and further physicochemical nature of N dopants to determine the mechanism of related redox reactions. Cheng group studied NPCs as pseudocapacitive electrodes both experimentally and theoretically, wherein N evolution upon electrochemical processes and intrinsic electronic structure of N‐species were thoroughly investigated.^31^ From first‐principles computations, they identified highly electron affinitive N‐X and less positively charged N‐6, generating well‐defined reaction mechanisms of redox pairs for N‐X and N‐6 as well as N‐5 and N‐6 (Equation 1), which are also supported by the ex‐situ x‐ray photoelectron spectroscopy (XPS) of NPC evolution. Also, first‐principles computations disclosed that both pyridinic and pyrrolic nitrogen can not only improve the accommodation of H atoms but also promote proton affiliation under the same distance compared with pristine graphene (**Figure** **2**a and 2b), demonstrating the possible improvement of both energy and power density.^16^

Thereby, EDLCs and pseudo‐capacitors of NPCs could be considerably enhanced by strengthening the physical and chemical interaction between the electro‐active sites and effective anions/cations in electrolytes. The possible reversible redox mechanisms of NPCs involved in acidic electrolytes are proposed as follows:^1^, ^30^


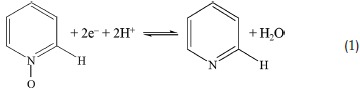



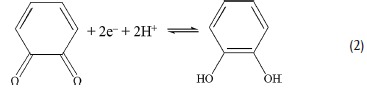


Apparently, the above redox reactions related to protons (or OH^−^/H_2_O), and accordingly electrochemical behaviors could be influenced by the properties of electrolytes (i.e., pH) and are also sensitive to the potential range (Pourbaix diagram). Based on the redox capability of functional groups, the suitable pH range as well as working electrochemical window for reactions should be carefully considered, which could lead to distinct pseudocapacitive behaviors in different medias.

### 2.2 Nitrogen Content, Porous Structure and Electrical Conductivity

There has no strict definition between the terminology of N‐doped and N‐rich carbons. In most cases, N‐doped carbons are derived from post‐treatment of well‐defined carbon materials, such as graphene or carbon nanotubes. Because of the stable sp^2^ conjugated structure, only a small amount of N could be doped into honeycomb lattice, end up being low N‐containing carbon. N in such materials is usually located at the edge, with pyridinic and pyrrolic N dominated structure, since fewer defects are available to react with N sources. Oppositely, N‐rich carbon materials are synthesized through nitridizing carbon precursor (such as gelatin) or directly pyrolyzing N‐containing materials (polyaniline or biomass), which will result in uniform distribution of N as well as multi‐species (N‐5, N‐6, N‐Q, and N‐X). The combined species would be more beneficial to achieve better performance as discussed.

Generally, the texture is one main factor that affects supercapacitive performance of carbon materials, wherein the N dopants can alter the properties. On one hand, a certain amount of electron‐donor N dopants along with induced structural defects/voids offer excellent electrical conductivity and transport paths, laying solid foundation for highly efficient electron and ion transfer. On the other hand, introduction of N species endows the electron donor characteristics of carbon layers and provides abundant electrochemically active sites for pseudocapacitve reactions. In this respect, traditionally the limited N doping level (1–3 wt%) usually hinders the exertion of positive effects, which only stops at increasing conductivity. In contrast, carbon materials with enriched N‐species are of great desire to release the restricted properties, usually showing better electrochemical performance.^32^, ^33^ For example, protein derived mesoporous N‐rich carbons (10.1 wt% N, sintering at 650 °C) reported by Li et al. exhibited a high capacitance of 390 F g^−1^, which is much larger than those of the obtained samples with lower N contents by sintering at higher temperatures (7.11 wt% N for 750 °C; 6.04 wt% N for 850 °C).^32^ Inconsistent phenomenon is reported in nonporous N‐enriched carbons obtained firstly by pyrolysis of polymerized ethylenediamine and carbon tetrachloride with further KOH activation.^34^ By adjusting the weight ratio of KOH, the resultant NPCs were harvested with different dopant contents, NPC‐X: NPC‐0.5 (10.08 wt% N), NPC‐1 (3.3 wt% N) and NPC‐2 (1.1 wt% N), wherein NPC‐1 with mildly doping achieves the best performance. The optimal combination of double‐layer capacitance and pseudocapacitance contributes to the largest specific capacitance of 363 F g^−1^ of NPC‐1, higher than those of NPC‐2 (303 F g^−1^) and NPC‐0.5 (247 F g^−1^). As one can see, supercapacitive performance usually shows the non‐linear correlation with the N content, which could be also found in other reports on NPCs.^20^, ^35^

Fundamentally, the capacitance of heteroatom‐involved carbon materials originates from both EDLCs relying on high surface area and pseudocapacitors induced by the heteroatom groups. Of course, it would prefer both surface area and N‐doping as possible, but in reality they are opposite due to the synthetic limitation and turn out trade‐off between each other. Normally, while the effective surface area of NPCs is enlarged when increasing the heat temperature or prolonging the heat time, the N‐content will decrease owing to the decomposition of functional species.^16^, ^18^, ^20^ Obviously in this scenario, though EDLCs can be enhanced by enlarging the specific surface area, pseudocapacitors will be significantly impaired. Therefore, the reported NPCs usually exhibited the highest capacitances at a moderate specific surface area and proper dopant content, testified by many cases such as N‐doped graphitic carbon nanocages,^36^ mesoporous N‐rich carbons,^32^ N‐doped ordered mesoporous carbon nanofiber arrays,^37^ N‐containing isoreticular metal‐organic framework derived NPCs,^38^ and heteroatom‐doped porous carbon flakes,^35^ showing that no simple linear relationship was found between the specific capacitance and the measured specific surface area as well as the content of N‐dopants in NPCs. Notably, for SCs with ultrafast charge‐discharge processes, the capacitance could be delivered only when surface is available for ions to access, making the external surface area more important than the overall one. Micropores with narrow crack but high depth have less contribution to the external surface, thus usually hindering or even blocking ion adsorption under high rates.^39^ Also, the pore shape with different geometries would be equipped with various ion transport kinetics.^40^ Finally, restacking of carbon nanosheets, particular graphene, is another factor that influences the full expression of theoretical capacitance. It is reported that the affordable capacitance of graphene materials could reach as high as 550 F g^−1^, but is largely suppressed by the easily restacking of graphene.^41^ Therefore, it is of great significance to avoid restacking when synthesizing N‐containing carbon nanosheets.

Basically, the higher temperature, the better electrical conductivity and the more porosity can be achieved, and the fewer N species would be maintained. All these trade‐off will give rise to the parabolic relationship between sintering temperature and performances,^35^, ^36^, ^37^, ^38^ wherein finding an optimum condition to obtain NPCs with a fairly large N content, a moderate surface area and good conductivity is a primary route to acquire high capacitance. Based on the literature search, the proper temperature is commonly suggested to be 650 to 850 °C. Within the correlation and mutually‐influenced factors, electrical conductivity, electrochemically‐active sites and porous structure, can be tuned by the N concentration, wherein the electrochemical behaviors are determined through all three complex dimensions.

### 2.3 Miscellaneous Electrochemical Behaviors in Different Media

Due to the diverse properties within different heteroatom functionalities, the electrochemical behaviors of NPCs depend on the applied electrolyte and are sensitive to the potential range.^1^ Introducing N‐containing functional groups makes the electrode interface more basic, especially for the carbon materials with large amount of N‐6 and N‐5 groups. Besides, N‐containing functionalities could increase the electronic density of carbon network and favor proton adsorption in acidic electrolytes, consequently bringing higher double‐layer capacitance in H_2_SO_4_ than that in KOH solutions. For example, pseudocapacitances derived from pyridine‐N and pyridone‐N are noticeably observed at a potential negative to 0.6 V vs. RHE (reversible hydrogen electrode) in 1 m H_2_SO_4_ solution (Figure 2c), while this pseudocapacitive contribution was weakened in 1 m KOH electrolyte (Figure 2d) due to the lack of protons for the basic functionalities to undergo redox reactions.^30^ The results demonstrate that the influences of N‐containing functional groups on the capacitive performance of carbons are strongly dependent on the electrolyte.

Electrode materials operated in neutral electrolytes can normally uphold a wider working potential range. Although nitrogen‐containing groups were claimed to have almost no benefits for pseudocapacitance in neutral and nonaqueous electrolytes,^17^ recent work has shown intriguing redox behaviors in these electrolytes. Though related reaction mechanisms remain unknown, the distinct redox peaks of nitrogen‐containing carbon located at ≈0.25 to ≈0.5 V vs. Ag/AgCl (Figure 2e) are supposed to stem from robust redox reactions at nitrogen‐associated defects that transform inert graphene‐like layered carbon into an electrochemically active substance without impairing the electrical conductivity.^8^ Notably, the capacitance of NPCs is always better in acidic or neutral environment than in alkaline electrolytes, which is mainly ascribed to the electron‐rich nature of N atoms to facially bind cations. Due to the enlarged voltage window, electrochemical performances can be considerably improved by using organic electrolytes. For example, symmetric SCs utilizing N‐containing carbon in 1 M Et_4_NBF_4_‐PC electrolyte can be operated reversibly in an extended working voltage up to 3.0 V (Figure 2f),^23^ which could boost the energy density to nearly 6 times as the obtained value in 6 M KOH solution with a voltage window of 0–1.0 V.

## 3 Progressive Developments of SC Configurations

Similar to all the energy storage devices, supercapacitors are assembled by the anode and cathode sandwiching a separator in‐between, wherein overall devices work in aqueous/non‐aqueous electrolytes. Classically, two categories are classified for supercapacitors, symmetric SCs with the same or similar electrodes at both ends and asymmetric ones with the configuration of different electrodes. Generally, energy density (*E*, W h kg^−1^) and power density (*P*, W kg^−1^) are both critically significant parameters for the evaluation of SCs regarding energy storage performance, which represent the storage capacity and power delivery, respectively.^42^, ^43^ Apparently, as the energy storage mechanism of SCs relies on merely surface charge storage, low energy density is always the greatest problem.

According to the mathematical expression of energy density (*E*, *E = 0.5 CV^2^*), where *E* is proportional to the specific capacitance (*C*, F g^−1^) and square of operating potential window (*V*, Volt), the optimization of electrode materials to achieve large specific capacitance and construction of SC configurations to extend working potentials are both effective ways to boost the energy density. Normally, the operating voltage of SCs relies on both electrochemically‐stable electrolytes and suitable combination of cathodes and anodes. Therefore, searching for high‐voltage electrolytes and optimal electrode pairs are crucial in enlarging the working voltage of SCs. Within the safe voltage range of electrolytes, the maximum voltage output depends on the difference of working windows between cathodes and anodes, as V_max_(cell) = V_max_(cathode) – V_min_(anode). Compared with conventional carbon materials, NPCs deliver largely enhanced capacitance owing to induced redox reactions by unique electron donor and/or acceptor effect of N‐doping, and meanwhile still maintain a wide electrochemical working window (−1−1 V in aqueous solutions) with robust structural integrity.^44^ Thereby, progressive development of NPCs is advancing the path in pursuing battery‐level energy density without scarifying the high‐power feature, where NPCs exert important functions in different SC configurations. In this section, we will summarize the state‐of‐the‐art investigations on different NPC‐based SCs and discuss their important contribution to achieve battery‐level energy density.

### 3.1 Aqueous Asymmetric SCs Based on NPCs

Benefiting from the distinct working potential ranges of the cathode and anode, the output potential would be noticeably enlarged by combining different materials to construct asymmetric SCs, thus greatly improving the energy density of assembled devices. Typically, pseudocapacitive cathodes (oxides) and high surface area carbon anodes (activated carbon, or graphene) are utilized in aqueous electrolytes. However, depend on the charge balance principle and the consequent mass ratio m_+_/m_−_ = (C_−_∆E_−_)/(C_+_∆E_+_), the required quantity of low‐capacitance anodes is always much excess of cathodes (normally over 3:1). Due to the low capacitance of double‐layer capacitive carbons (100–200 F g^−1^), the overall performance of full SCs are restricted below this value. Therefore, exploring high‐performance anode materials is always one of urgent goals in the development of high‐energy aqueous asymmetric SCs.

**Figure 3:**
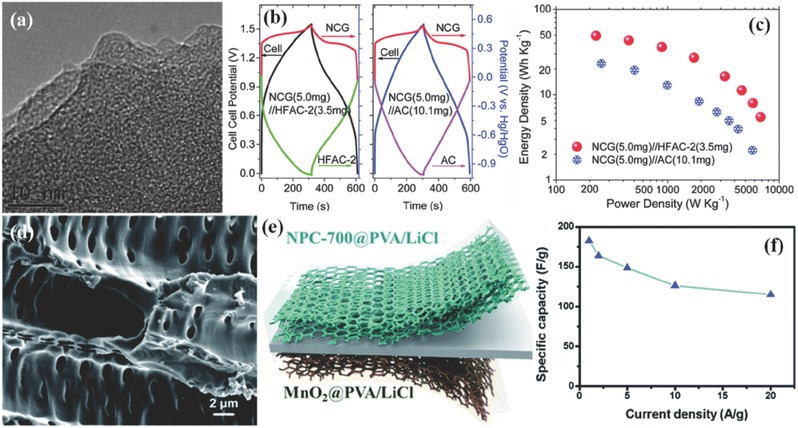
a) High resolution transmission electron microscope (TEM) of HFAC. b) Galvanostatic charge/discharge curves of the two asymmetric cells at 0.5 A g^−1^, and the in situ tracked variation of the potential in each individual electrode. c) Ragone plots of energy density versus power density for the asymmetric cells. a–c) Reproduced with permission.^20^ Copyright 2014, Royal Society of Chemistry. d) A SEM image of the NPC material. e) Schematic diagram of the fabricated MnO_2_//NPC‐700 asymmetric SC, and (f) corresponding specific capacitance as a function of current density. d–f) Reproduced with permission.^45^ Copyright 2016, Royal Society of Chemistry.

At this point, N‐rich carbon frameworks are verified to improve the charge storage capability by furnishing more electrochemically active sites for redox reactions. For instance, Mitlin group prepared highly functionalized activated carbons (HFACs) from egg white,^20^ and the obtained monolithic carbons are both highly nitrogen rich (4.1–7.6 wt.%) and large microporous (88 vol% micropores, surface area up to 1405 m^2^ g^−1^, **Figure** **3**a). In the optimized state, the electrode demonstrates a remarkably large capacitance of ≈525 F g^−1^ in 1 M KOH, which could be productively assembled into asymmetric SCs as the anode to pair NiCo_2_O_4_/graphene (NCG) with similar mass (Figure 3b), leading to a huge improvement over traditional AC‐based devices in terms of two‐fold gravimetric energy density (Figure 3c) and four‐fold volumetric performance.

Nitrogen‐rich porous carbons with permeable defect‐related porosity possess abundant active sites in situ created at pores, which fortify the charge storage within microporosity. An N‐rich nanoporous carbon (NPC, Figure 3d) with large surface area and abundant micro‐/meso‐ pores was prepared by Xiao et al. via a facile pyrolysis process of shaddock peel.^45^ In addition to the energy storage contribution of micropores, the presence of mesopores plays a critical role in ion transport and electrolyte diffusion. Therein, superb rate capability with high cycle stability is achieved within alkaline electrolytes. Further all‐solid‐state asymmetric SC devices highlighted unique anode‐cathode balance (mass ratio of NPC to MnO_2_ is 1.25:1) as well as the corresponding ultrahigh specific energy of 82.1 W h kg^−1^ (Figure 3e, and 3f).

### 3.2 Novel Symmetric SCs Based on NPCs

#### 3.2.1 Novel Aqueous Symmetric SCs

**Figure 4:**
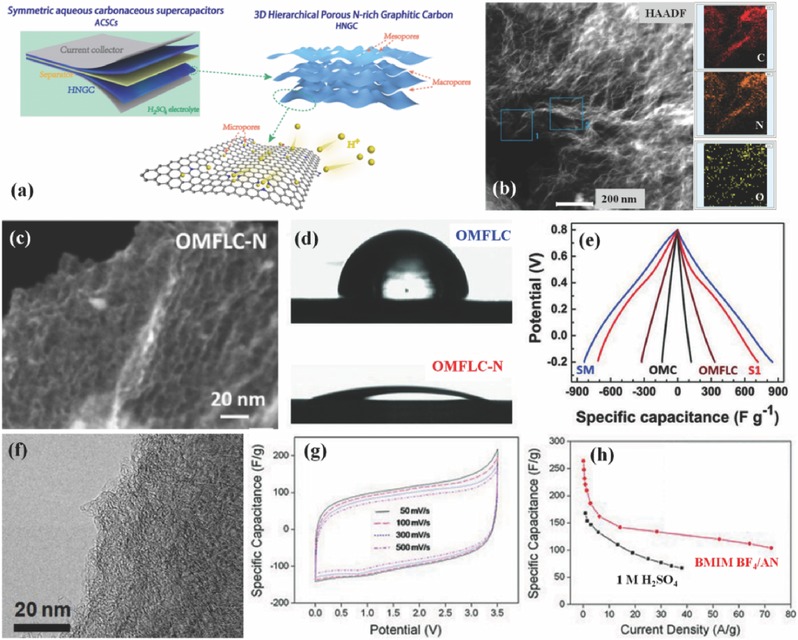
a) Illustrated protocol of aqueous symmetric SCs fabricated with two NPC electrodes. b) A dark field scanning TEM image along with element mapping of C, N and O. a,b) Reproduced with permission.^16^ Copyright 2015, Royal Society of Chemistry. c) A dark field scanning TEM image of OMFLC‐N. d) Wetting angles of 0.5 m H_2_SO_4_ droplet on OMFLC (85°) and OMFLC‐N (S1) (21°) substrates. e) Galvanostatic charge‐discharge test at 1.0 A g^−1^ from the first cycle. c–e) Reproduced with permission.^8^ Copyright 2015, AAAS. f) TEM image of the H‐CMNs. g) CV curves of the H‐CMNs at different scan rates over a potential range from 0 to 3.5 V in the BMIM BF_4_/AN electrolyte. h) Specific capacitances at various current densities measured in organic and aqueous electrolytes. f–g) Reproduced with permission.^24^

Symmetric SCs are traditionally trapped in low operating voltage (V) and further low energy density. By comparison, reported carbon//carbon symmetric configurations in aqueous solutions could achieve much wider electrochemical windows of 1.2–1.6 V by benefiting from enlarged over‐potential,^44^, ^46^ which can almost rival most asymmetric counterparts (1.4–1.8 V). However, because of the mere physical charge storage (EDLCs), non‐doped carbon‐based symmetric SCs demonstrated unsatisfying energy storage capability. Except for inheriting the wide voltage window (−1.0–1.0 V) and robust structural integrity of carbon materials,^44^ NPCs could also deliver noticeably enhanced capacitance owing to induced redox reactions and thus largely compensate the low‐capacity drawback, which is suitable for constructing novel symmetric SCs with both high operation voltage and high‐level capacitance. For example, Yang et al. prepared hierarchical porous N‐rich graphitic carbon (**Figure** **4**a and 4b) with beneficial multi‐porous structures and especially copious N‐containing groups.^16^ The functional carbon material delivers ultrahigh capacitance of over 710 F g^−1^ in 1 M H_2_SO_4_ aqueous electrolyte, which also facilitates symmetric SCs a high operating voltage of 1.5 V and outstanding battery‐level specific energy of over 75 W h kg^−1^ at 1500 W kg^−1^ as well as superb volumetric indices. Moreover, even bearing a mass loading of above 10 mg cm^−2^, the functional carbon still maintains exceptional capacitance 3−4 times of pristine carbon and represents typical supercapacitive behaviors synchronizing both double layer and redox capacitive features.

More recently, Lin et al. have also fabricated a nitrogen‐rich ordered mesoporous few‐layer carbon (OMFLC‐N, Figure 4c) with higher capacitance in acidic H_2_SO_4_ (855 F g^−1^) and neutral Li_2_SO_4_ (780 F g^−1^) electrolytes.^8^ Nitrogen profoundly altered physical properties of carbon frameworks into surface hydrophilic (Figure 4d) and induced defect sites for robust faradic reactions, making immense contribution to capacitance enhancement (Figure 4e) that transforms inert carbon into an electrochemically active substance without impairing the intrinsic conductivity. The operating voltage of as‐fabricated symmetric SCs could achieve as high voltage as 1.6 V in 2 M Li_2_SO_4_ electrolyte, wherein a maximum energy of 63.0 W h kg^−1^ as well as a maximum specific power of 44.0 kW kg^−1^ (with a fast discharging time of 5.7 s) were delivered. Obviously, from both aforementioned cases, NPC‐based symmetric SCs hold great promise in that high energy density is concurrently achieved along with high power delivery, making the carbonaceous symmetric SCs potentially competitive against batteries, such as lead‐acid batteries, nickel metal hydride batteries, and even some lithium ion batteries.

#### 3.2.2 High Voltage Non‐Aqueous Symmetric SCs

As stated, non‐aqueous electrolytes, free from the water splitting restriction, are beneficial for high‐voltage applications. Thus, non‐aqueous SC devices could proffer a favorable operating voltage above 3.0 V, considerably improving energy storage performance. Jin group conducted a comprehensive study on hierarchically porous carbon nanofibers which contains plentiful electroactive heteroatoms,^24^ especially nitrogen‐containing textures after the treatment with melamine (9.1 at.% nitrogen). Symmetric SCs were assembled by adopting this functional NPC in the BMIM BF_4_/AN electrolyte, and the full cells were reversibly operated over expansive potential range from 0 to 3.0 V, displaying a specific energy of 113 W h kg^−1^. Similarly, they also reported carbon‐based microporous nanoplates containing numerous heteroatoms (H‐CMNs, 5.1 wt.% N) from regenerated silk proteins. As presented in Figure 4f– 4h, the operating voltage of corresponding symmetric SCs in the BMIM BF_4_/AN electrolyte is as high as 3.5 V and leads to a remarkable energy density of 133 W h kg^−1^, which is nearly three fold of the specific energy of aqueous supercapacitors. To achieve symmetric SCs with the integration of high energy and power density, heteroatom‐containing carbon materials should realize both high capacitance and good rate capability by acquiring tailor‐made surface chemical, electronic and porous properties.

Undeniably, a high working potential is the outstanding advantage for asymmetric SCs. But for capacitance parts, both electrodes (1/C_cell_ = 1/C_+_ + 1/C_−_) are important, wherein specific capacitance of full cells is determined and limited by the lowest one. Also, charge balance between two sides further calls for mass matching of two electrodes in asymmetric configurations (m_+_/m_−_ = (C_−_∆E_−_)/(C_+_∆E_+_)). Adding weight on the low capacity side will further diminish the overall capacitance. In this respect, assembling symmetric devices with the same electrodes at both sides could not only simplify fabrication procedures, but also maximize the performance of each electrode by avoiding the commonly observed energy/power imbalance in asymmetric counterparts. Therefore, owning a fairly large operation voltage, novel symmetric SCs constructed with high‐capacitance NPCs show competitive advantages over asymmetric counterparts.

### 3.3 Emerging Non‐Aqueous Hybrid SCs Based on NPCs

**Figure 5:**
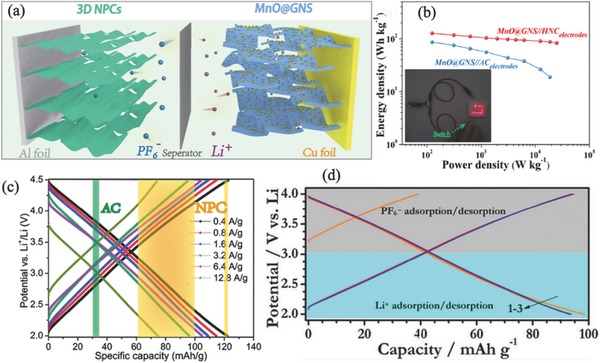
a) Schematic illustration of Li‐HSC composed of MnO@GNS//HNC. b) Ragone plots of the hybrid cell, and CV curves. a,b) Reproduced with permission.^25^ c) Galvanostatic charge‐discharge curves of NPC at different current densities as well as the reported capacity of AC (green region) and NPC materials (yellow region). Reproduced with permission.^27^ Copyright 2016, Royal Society of Chemistry. d) Charge‐discharge behaviors of NPCs at different voltage ranges. Reproduced with permission.^54^

High energy lithium‐ion batteries (LIBs) and high power SCs rely on different fundamental working principles: bulk vs. surface ion diffusion. Principally hybridization between SCs and LIBs, termed as Li‐ion hybrid SCs (Li‐HSCs) or Li‐ion capacitors, has been explored as an emerging hybrid technology in recent years.^47^, ^48^ Typically, Li‐HSCs are composed of a capacitor type cathode and a battery type anode in suitable organic electrolytes, wherein the anode stores cations (such as Li^+^) through bulk‐phase reactions and cathode adsorbs anions via surface capacitive mechanism (**Figure** **5**a). Under this configuration, the anode enhances overall energy storage capability while the cathode builds up the fast kinetics, simultaneously leading to high performances. At the very start, conventional carbon materials with large surface area, such as AC, have been utilized as cathodes, wherein the low stored charge from merely EDLCs undermines full expression of high energy feature. In this scenario, recent reports on NPCs have demonstrated considerable improvement of performance and efficient circumvention on current challenges, highlighting the highly promise as cathodes for high‐performance hybrid SCs.

According to the capacitance‐determining rules, the overall energy density of Li‐HSCs are determined by cathodes, but the low capacitance of commercial AC (≈30 mA h g^−1^) is still a crucial issue to be overcome.^49^, ^50^ Physically optimizing porosity could enhance capacitance, wherein porous structures with large external surface area facilitate effective ion adsorption.^51^ For example, tailored active carbon prepared by Jain et al. could achieve 60% mesoporosity and high performance of over 60 mA h g^−1^. On the other hand, chemical doping is also conducive to build favorable porous structure, which can further offer supplementary electrochemical reactions to improve surface charge storage.^52^, ^53^ For example, Yang et al. artfully engineered a high‐power Li‐HSCs device utilizing a hierarchical N‐rich carbon (HNC) as the cathode to pair an ultrafine MnO@GNS anode (Figure 5a). Owing to abundant N‐induced defects, NPC cathodes are endowed with interconnected porous structure, both leading to almost twice higher capacitance than commercial AC.^25^ Combining with fast surface‐enhanced lithium storage at anodes, the hybrid system achieved an impressive energy storage of 127 W h kg^−1^ (Figure 5b), and as high as 83.25 W h kg^−1^ even at a battery‐inaccessible specific power of 25 kW kg^−1^ (with rapid charging time of 8 s), successfully integrating high power‐energy delivery. Particularly, cyclic voltammetry of the hybrid cell show near‐rectangular shapes with the identification of slight humps, indicating electrochemical behaviors involving both redox and capacitive reactions.

Similarly, 3D carbon nanosheets (CNS) developed by Mitlin group attained a high capacity of 70 mA h g^−1^ at 0.1 A g^−1^ as the cathode.^55^ Although reactions regarding anion adsorption on N‐species remain unclear, N‐doping is clearly beneficial to the capacitance promotion of cathodes for Li‐HSCs. Very recently, Li et al. have prepared highly porous N‐doped activated carbons by employing agricultural corncob as a precursor, achieving superb and stable capacity of ≈120 mA h g^−1^ (2.0–4.5 V vs. Li/Li^+^).^27^ The capacity of NPC materials could be boosted up to 4 times as commercial carbons (30–35 mA h g^−1^, Figure 5c). When increasing the N content to 2.97 wt%, both the specific capacity and cycling stability are enhanced, highlighting the importance of N‐species for high performance. Owing to the bifunctional nature, Li ion adsorption (typically 2–3 V vs. Li/Li^+^) is also constructive for NPC materials,^54^ wherein electron donating N‐species could improve the performance accordingly (Figure 5d). In the case of paring prelithiation anodes, such Li adsorption at cathodes could largely increase the performance of device entirety in terms of energy and power density.^56^

As for Li‐HSCs, high‐voltage hybrid devices could be achieved by matching low‐potential negative electrodes with positive counterparts. An anode with a low Li^+^ intercalation potential (such as graphite) coupled with a cathode adsorbing anions, forming a hybrid configuration, will definitely gain a high voltage window for full cells. For instance, by coupling a 3D interconnected TiC nanoparticle chain anode with a hierarchical porous nitrogen‐doped carbon cathode, Wang et al. fabricated a hybrid device with a high working voltage window up to 4.5 V in an ordinary organic electrolyte (LiPF_6_ in carbonate solution).^53^ Besides, adopting high‐voltage electrolytes could push the adsorption of anions to high potentials in the cathode, bringing in desirable output of full cells.

## 4 Conclusion and Prospective

In this new era of energy storage, nitrogen‐rich porous carbon materials offer great opportunities to promote SCs towards battery‐level energy density, demonstrating many breakthroughs in broad applications ranging from asymmetric to symmetric and hybrid SCs. With favorably tuned physicochemical properties through nitrogen functionalities, NPC materials always manifest simultaneous enhancement in electrical conductivity, porous structures as well as electrochemically‐active sites compared with common carbon materials, which further results in huge advance on capacitive performance by introducing redox reactions at/near carbon surface. Further, NPCs are equipped with versatile properties as both cathodes and anodes, showing intriguing flexibility and superb performance in a wide electrochemical potential regions for constructing high voltage devices. Notably, according to the role of different N‐species playing in electrolytes, tailored dopants regarding doping types and contents are required to balance conductivity‐porosity‐active sites trade‐off and attain optimal performance with the matched operation environment. Specifically, NPCs exert different functions as a promoter in SC devices. As for asymmetric configurations, NPCs with high capacitance tactfully reduce the cathode‐anode mass imbalance and further improve energy‐power delivery. It also revives the symmetric SCs by exploiting the redox reactions, and scaling the energy to a new height. Building at both sides of hybrid SCs, NPCs establish a bridge to solve capacity and kinetics mismatches between battery‐type and the capacitor‐type electrodes, leading to a more consistent energy storage performance than commercial carbons.

It has reached a consensus that NPCs are beneficial for capacitive performance by taking advantage of intrinsic electron donor nature. However, due to the complexity of nitrogen dopants, the fundamental reaction mechanism of specific species remains unclear and the investigation of physicochemical properties are still at the preliminary stage, although assuring the active sites of electrochemical processes is imperative for guiding high‐performance SCs. Moreover, instead of randomly functionalizing, green and facile synthetic technologies founded on molecule assemblies are still desperately needed for controllable heteroatom‐doped carbon materials, especially achieving the controllable doping of heteroatom‐species and relative contents. Besides, it is worth mentioning that the incorporation of N and other dopants (such as S, P, F, and B) into the carbon framework at the same time is now getting more popular in carbon‐research communities, which could considerably improve electrochemical performance due to the synergistic effects of codoping heteroatoms. Selectively choosing a proper combination of co‐dopants should be based on the specific requirements of different applications. For example, the expanded carbon interlayer results from S‐doping facilitates the adsorption of larger electrolyte ions; highly electronegative F functional groups will strongly enhance polarization and refine pore structures/surfaces; formation of different N configurations could be modulated by incorporation of P into the carbons. Moreover, P doping is recommended when the application is sensitive to O‐containing groups or wetting properties are required. At last, although some literature identified the positive impacts and synergistic effects of surface co‐doping on the improvement in capacitive performance, the detailed mechanisms and exact roles of different surface groups still need further investigations. Therefore in the future research, mechanism studies on electrochemistry as well as methodology development on synthesis require simultaneous efforts to promote NPC‐based SCs. With unremitting endeavor, we believed that deeper insight will be uncovered and versatile high performance SCs would stand out among current energy technologies.
